# The Role of High-Risk Cytogenetics in Acute Kidney Injury of Newly Diagnosed Multiple Myeloma: A Cohort Study

**DOI:** 10.3390/ijms26136108

**Published:** 2025-06-25

**Authors:** Carolina Branco, Manuel Silva, Natacha Rodrigues, Joana Vieira, João Forjaz Lacerda, José António Lopes

**Affiliations:** 1 Division of Nephrology and Renal Transplantation, Unidade Local de Saúde Santa Maria, 1649-035 Lisbon, Portugal; carolinagbranco@hotmail.com (C.B.); manueljoaosantoslopessilva@gmail.com (M.S.); natachajrodrigues@gmail.com (N.R.); 2 Faculdade de Medicina, Universidade de Lisboa, 1649-028 Lisbon, Portugal; jflacerda@chln.min-saude.pt; 3 Division of Hematology, Unidade Local de Saúde Santa Maria, 1649-035 Lisbon, Portugal; joana.r.vieira@ulssm.min-saude.pt; 4 Gulbenkian Institute for Molecular Medicine (GIMM), 1649-028 Lisbon, Portugal

**Keywords:** high-risk cytogenetics, acute kidney injury, multiple myeloma, epidemiology

## Abstract

Multiple myeloma (MM) is frequently associated with cytogenetic abnormalities, with high-risk cytogenetics linked to poorer survival. Acute kidney injury (AKI) is common in MM, but its relationship with high-risk cytogenetics remains underexplored. This study aimed to assess the association between high-risk cytogenetics and AKI in newly diagnosed MM patients and to evaluate their impact on overall survival, relapse-free survival, and progression to chronic kidney disease (CKD) in the first two years after diagnosis. We conducted a single-center retrospective cohort study including patients newly diagnosed with MM between 2018 and 2022. We enrolled 122 patients. AKI was observed in 36.9% of patients, rising to 62.3% among those with high-risk cytogenetics. High-risk cytogenetics (OR: 3.32; 95% CI: 1.17–6.40; *p* = 0.024), CKD (OR: 9.14; 95% CI: 2.92–18.65; *p* < 0.001), kappa free light chains, hypercalcemia, difference in free light chain (dFLC), and bone marrow plasmocyte percentage were independently associated with AKI. Both AKI (HR: 2.71; 95% CI: 1.18–6.23; *p* = 0.019) and high-risk cytogenetics (HR: 3.33; 95% CI: 1.13–9.76; *p* = 0.029) were independently associated with lower overall survival. Among survivors without prior CKD, progression to CKD was higher in those with AKI (30.7% vs. 9.3%; *p* = 0.041). High-risk cytogenetics were significantly associated with AKI in MM patients. Both factors independently predict worse survival and increased risk of CKD progression.

## 1. Introduction

Multiple myeloma is a clonal plasma cell malignancy and the second most common hematological neoplasm, with its incidence increasing over the past 25 years [1]. It is a highly heterogeneous disease in terms of cytogenetics, clinical presentation, therapeutic response, and overall survival. Cytogenetic abnormalities have been studied since the early 2000s and appear to contribute, at least in part, to the variability in clinical features and outcomes [2].

Primary genetic events, occurring during the monoclonal gammopathy of undetermined significance (MGUS) phase, primarily involve translocations (mainly with chromosome 14) and trisomies (particularly of odd-numbered chromosomes such as 3, 5, 7, 9, 11, 15, 19, and 21), seen in about 30% and 40% of patients, respectively [1,3,4,5]. While trisomies 3 or 5 are associated with improved survival, trisomy 21 is linked to poorer outcomes [6]. Secondary genetic events, arising during the multiple myeloma phase, include chromosomal translocations, copy number variations, and single-nucleotide variants [3,4]. Monosomy and the deletion of chromosome 13, along with 17p deletion, are common and often correlate with more aggressive disease [7]. The amplification of 1q, observed in roughly 40% of patients, is also associated with an unfavorable prognosis [1,8].

Nowadays, high-risk cytogenetic abnormalities, commonly referred to as high-risk cytogenetics, are the most widely recognized prognostic indicators of poor outcomes in multiple myeloma, being associated with reduced disease-free survival and lower overall survival. Clinical parameters such as serum lactate dehydrogenase, serum albumin, and serum β2-microglobulin, which form the basis of the revised International Staging System (R-ISS) for multiple myeloma, are also associated with poor prognosis [9,10]. More recently, R-ISS has been updated with the introduction of R2-ISS, which incorporates the role of 1q21 gain in prognosis and the coexistence of more than one high-risk cytogenetic abnormality [11].

Renal involvement in multiple myeloma is knowingly diverse, ranging from disorders directly caused by monoclonal immunoglobulin deposition to other pathophysiological mechanisms (e.g., damage linked to tumor burden or treatment-related adverse effects) [12]. Each condition warrants a tailored and timely therapeutic approach, as the reversal of renal injury has been associated with improved overall survival, highlighting the need for the early identification of at-risk individuals and the implementation of effective preventive measures [12,13,14]. Renal impairment associated with multiple myeloma is diagnosed in at least 50% of patients during the course of the disease [12]. The impact of AKI in patients with multiple myeloma has been studied at different stages of the disease, such as at diagnosis or after Hematopoietic Stem Cell Transplantation (HSCT) [15,16]. However, there is still limited knowledge regarding the implications of high-risk cytogenetics on kidney function.

The purpose of this study is to evaluate the association between the presence of high-risk cytogenetics and AKI in newly diagnosed multiple myeloma patients; to determine the impact of AKI on overall survival and relapse-free survival during the first two years after diagnosis; and to investigate the impact of AKI on the progression to Chronic Kidney Disease (CKD) in survivors without a previous diagnosis of CKD two years after the initial diagnosis of multiple myeloma.

## 2. Results

**Figure 1:**
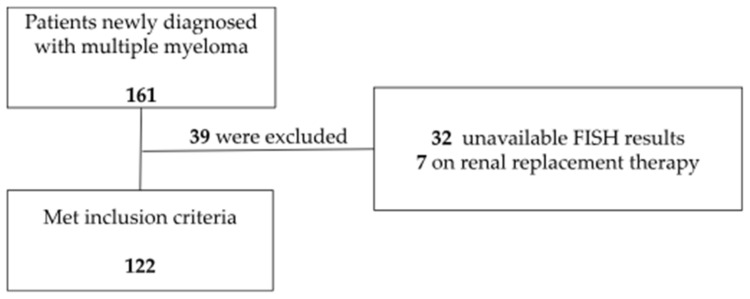
Flowchart of patients included in the cohort study.

One hundred and sixty-one patients with newly diagnosed multiple myeloma were referred to the Haematology and Bone Marrow Transplantation Department of ULS-SM between January 2018 and December 2022. FISH results were unavailable for thirty-two patients. Seven patients had been receiving renal replacement therapy due to causes unrelated to multiple myeloma. Therefore, 122 patients were eligible for inclusion in the study (Figure 1). The patients’ baseline characteristics and multiple myeloma-related information are presented in Table 1.

### 2.1. AKI at Diagnosis

The incidence of AKI and moderate-to-severe AKI at diagnosis was 36.9% (45 patients) and 19.7% (24 patients), respectively. High-risk cytogenetics were present in 62.3% of patients with AKI at diagnosis, compared to 37.6% of those without AKI at diagnosis. The univariable analysis of AKI in newly diagnosed multiple myeloma patients is presented in Table 2.

In this analysis, variables associated with AKI included a prior diagnosis of CKD, lambda light chain involvement, hypercalcemia, high-risk cytogenetics, dFLC levels, β2-microglobulin, LDH, and bone marrow plasmocyte percentage.

In our multivariable model of newly diagnosed multiple myeloma patients, we found independent associations with high-risk cytogenetics (OR: 3.32; 95% CI: 1.17–6.40; *p* = 0.024), CKD (OR: 9.14; 95% CI: 2.92–18.65; *p* < 0.001), lambda light chain involvement (OR: 3.70; 95% CI: 1.31–6.45; *p* = 0.013), hypercalcemia (OR: 3.17; 95% CI: 1.50–5.36; *p* < 0.001), dFLC (OR: 1.01; 95% CI: 1.00–1.02; *p* = 0.015), and bone marrow plasmocyte percentage (OR: 1.02; 95% CI: 1.01–1.02; *p* = 0.010) (Table 3).

### 2.2. The Impact of AKI on Overall Survival

**Figure 2:**
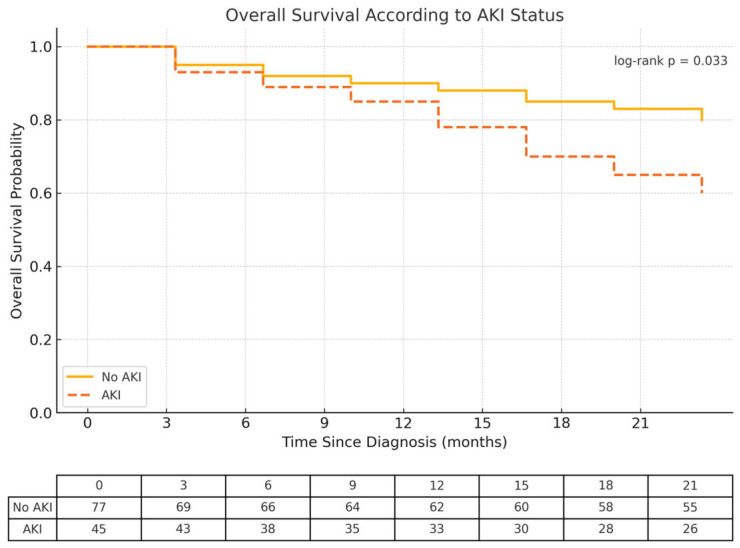
Overall survival considering AKI in the first 3 years after the diagnosis. AKI—acute kidney injury.

By the end of the 2-year follow-up period, overall survival was lower in patients who presented with AKI at the time of diagnosis compared to those without AKI (log-rank test for equality of survivor functions: *p* = 0.033) (Figure 2). A total of 31 patients (25.4%) had died—37.7% of those with AKI versus 18.2% of those without AKI.

In the univariable analysis, AKI was associated with lower overall survival. Other variables associated with reduced overall survival included high-risk cytogenetics, age, CKD, heart disease, secondary amyloidosis, dFLC, β2-microglobulin, and treatment with proteasome inhibitors (Table 4).

In the multivariable analysis, AKI was independently associated with lower overall survival (HR: 2.71; 95% CI: 1.18–6.23; *p* = 0.019). Other variables independently associated with reduced overall survival included high-risk cytogenetics (HR: 3.33; 95% CI: 1.13–9.76; *p* = 0.029), diabetes mellitus (HR: 1.99; 95% CI: 1.59–4.44; *p* = 0.026), and heart disease (HR: 3.70; 95% CI: 1.75–7.83; *p* = 0.001) (Table 5).

### 2.3. The Impact of AKI on Relapse-Free Survival

The cumulative incidence of relapse was 26.4% by the end of the 2-year follow-up period—22.7% in patients with AKI versus 31.6% in those without AKI, with no statistically significant difference between groups (log-rank test for equality of survivor functions: *p* = 0.068). No statistically significant association was found between AKI and decreased disease-free survival.

### 2.4. The Impact of AKI on the Progression to CKD

Sixty-seven patients did not have CKD at the time of diagnosis and were still alive two years after the initial diagnosis of multiple myeloma. By the end of the 2-year period, 13.4% of these patients had developed CKD—30.7% of those with AKI versus 9.3% of those without AKI (two-proportion *z*-test: *p* = 0.041). A statistically significant association was found between AKI and progression to CKD two years after the initial diagnosis of multiple myeloma.

## 3. Discussion

Renal injury is both a diagnostic feature and a common complication of multiple myeloma [12,13,17,18,19]. Many studies on renal injury in multiple myeloma have used alternative definitions, such as renal impairment (defined by an estimated glomerular filtration rate < 40 mL/min/1.73 m^2^) or renal failure (defined by serum creatinine levels > 2 mg/dL), rather than AKI. The incidence of these conditions has ranged from 19% to 50%. Knudsen et al. (1994) [20] reported a 31% incidence of renal failure, Kyle et al. (2003) [21] found a 19% incidence of renal failure, Yadav et al. (2016) [22] observed a 50% incidence of renal impairment, and Eleutherakis-Papaiakovou et al. (2016) [23] described a 21% incidence of renal failure.

Despite these valid definitions of renal injury, we consider AKI to be extremely important, considering its well-established association with worse prognoses in several other clinical settings. The most recent definition of AKI was proposed in 2012 by the KDIGO [24]. The KDIGO classification resulted from the merging of the former classifications: Risk, Injury, Failure, Loss of Kidney Function, and End-Stage Kidney Disease (RIFLE) in 2004 [25] and the Acute Kidney Injury Network (AKIN) in 2007 [26]. This classification has been adopted worldwide to standardize the concept of AKI within the scientific community.

The incidence of AKI according to the KDIGO classification in our population of newly diagnosed multiple myeloma patients was 36.9% (19.7% when considering moderate-to-severe AKI).

In our cohort, the presence of high-risk cytogenetics was independently associated with a 3.32-fold higher risk of AKI. This finding suggests a pathogenic role of these cytogenetic abnormalities in the process of organ damage, which may be related to more aggressive disease or other factors that warrant further investigation. Recently, it has been suggested that the presence of certain translocations may be associated with an increased frequency of renal impairment. Abdallah et al. (2020) [27] found that patients with t(14;16)(q23;q23), t(6;14)(q25;q32), and t(14;20)(q32;q11) had a higher proportion of renal dysfunction, defined as creatinine levels ≥ 2 mg/dL. Greenberg et al. (2014) [28] showed that half of the patients with renal failure exhibited translocations without trisomies, although these translocations were present in only one-third of their study population—particularly t(14;16), which accounted for 13.5% of patients with renal failure as the only myeloma-defining event.

Along with our results, this growing evidence of linkage between AKI and high-risk cytogenetics highlights the need to better understand the pathophysiological rationale in order to explore the potential utility of cytogenetic data for early AKI risk stratification.

We identified other risk factors for AKI in addition to high-risk cytogenetics, such as a previous diagnosis of CKD, hypercalcemia, higher levels of dFLC, monoclonal light chain kappa, and a higher percentage of monoclonal plasma cells in the bone marrow.

CKD is a known risk factor for AKI in several clinical scenarios [29,30,31]. CKD results in a state of constant relative hypoxia with reduced numbers of peritubular capillaries, increased collagen deposition, myofibroblast proliferation, increased activation of the renin-angiotensin system, and reduced numbers of glomeruli, leading to hyperfiltration and higher tubular oxygen consumption in the corresponding tubules [32]. These factors, combined with chronic leukocyte infiltration and the pro-inflammatory environment of CKD, result in reduced renal reserve and maladaptation, the loss of autoregulation, and abnormal vasodilation, which create ideal conditions for enhanced susceptibility to AKI. Hypercalcemia, often seen in cases with extensive bone involvement, can induce AKI through several mechanisms, including its vasoconstrictor and polyuric effects, as well as being a significant contributor to lithiasis and medullary injury due to calcium deposition [33,34]. Serum dFLC reflects the level of serum light chain proteins produced by plasma cells. The increasing risk of AKI as dFLC rises emphasizes its role as a predictor of AKI, with several guidelines suggesting that a serum FLC concentration > 150 mg/dL or a dFLC > 100 (especially when associated with >1 g/d proteinuria and <10% albuminuria) may be sufficient to diagnose light chain cast nephropathy, thus obviating the need for a kidney biopsy [33,34,35,36].

The association between AKI and a higher percentage of bone marrow plasma cells may relate to a higher burden and/or more aggressive disease. Patients with kappa light chains were at higher risk for AKI compared to those with lambda light chains. Due to their electrophysical characteristics, kappa light chains are more frequently observed in cast nephropathy, while lambda light chains are more commonly associated with amyloidosis. The known higher incidence of cast nephropathy compared to amyloidosis may explain this finding.

In our study, both AKI (with a 2.71-fold higher risk) and high-risk cytogenetics (with a 3.33-fold higher risk) were independently associated with lower overall survival in the first two years after the diagnosis of multiple myeloma. Lower overall survival has consistently been associated with both AKI [12,13,17,18,37] and high-risk cytogenetics [1,18,19]. By including both AKI and high-risk cytogenetics in our multivariable model and demonstrating independent associations with overall survival for both, we can infer that the impact of AKI on overall survival is not solely attributable to its association with high-risk cytogenetics.

It is interesting to examine the overall survival curves in patients with AKI versus those without AKI and observe that they tend to diverge, particularly after the first year. This suggests that the likely impact of AKI on overall survival is not related to the initial episode of AKI but rather to the long-term consequences of this complication.

We found a statistically significant association between AKI and progression to CKD two years after the initial diagnosis of multiple myeloma in survivors without a previous diagnosis of CKD (30.7% vs. 9.3%, *p* = 0.041). The analysis of CKD progression excluded patients who died within two years, which we acknowledge as the introduction of a survivorship bias, yet it confers an important finding for physicians during long-term follow-up and reinforces the importance of multidisciplinary follow-up for these patients.

We believe that these findings reinforce the need to consider AKI not as an isolated event but as a potential inflection point in the long-term renal trajectory of patients with multiple myeloma. Given the association we observed between AKI and subsequent CKD, as well as its apparent impact on overall survival beyond the first year, our findings may serve as a basis for future prospective studies aimed at early nephrological risk stratification. In particular, the integration of cytogenetic data with renal outcomes could help identify patient subgroups that might benefit from closer follow-up, early intervention, or tailored therapeutic strategies. Such hypothesis-generating work may ultimately contribute to improving renal and overall outcomes in this population.

The primary limitation of this study arises from its retrospective design and single-center setting, which may limit the generalizability of the findings and data availability. Due to this aspect, it was not possible to assess urinary output, another criterion for AKI diagnosis. Our small sample size did not allow for the evaluation of the potential influence of each specific cytogenetic abnormality, and so it was not possible to interpret the biological heterogeneity of different cytogenetic lesions.

Despite these limitations, to the best of our knowledge, this is the first study to assess the impact of high-risk cytogenetic abnormalities in multiple myeloma-associated AKI. Given the relatively recent availability of multiple myeloma cytogenetic assessments in clinical practice, we believe our results are relevant and warrant further investigation.

## 4. Materials and Methods

### 4.1. Study Design, Population, and Data Collection

We conducted a single-center retrospective cohort study, conducted in accordance with the STROBE guidelines (Strengthening the Reporting of Observational Studies in Epidemiology) [38]. The STROBE checklist is available in the Supplementary Materials for consultation. We included patients with newly diagnosed multiple myeloma referred to the Haematology and Bone Marrow Transplantation Department of Unidade Local de Saúde Santa Maria (ULS-SM) between January 2018 and December 2022 who had been tested for the presence of high-risk cytogenetic abnormalities.

We excluded patients whose interphase fluorescence in situ hybridization (FISH) results were inconclusive due to insufficient samples, patients with CKD who were on dialysis for renal disease unrelated to multiple myeloma, and patients with other active solid or hematologic cancers.

Data were collected from individual electronic clinical records and included patients’ demographic characteristics (age, gender, race) and medical history (CKD, hypertension, diabetes mellitus, heart disease). It also encompassed multiple myeloma characterization at diagnosis (myeloma isotype, the presence of extramedullary plasmacytoma and associated amyloidosis, the presence of high-risk cytogenetics, the measurement of M component, β2-microglobulin, lactate dehydrogenase, dFLC, free light chain ratio, percentage of monoclonal plasmocytes in bone marrow, the presence of hypercalcemia, albumin levels, AKI and AKI stage), undergone treatments, and subsequent events (treatment with or without proteasome inhibitors, eligibility for autologous HSCT, time to relapse, time to death from all causes, renal function at the end of the follow-up period). Missing data for all variables represented less than 10%; therefore, no imputation techniques were used.

All patients were followed until death or censored at 24 months after diagnosis.

### 4.2. Definitions

FISH results considered as high-risk cytogenetics included the detection of t(4;14), t(14;16), del(17p), 1q gain [gain(1q)], and 1p deletion [del(1p)]. Our laboratory follows the recommendations set by the European Myeloma Network (EMN) FISH workshop [39], with a cut-off level for considering a positive result of 10% for IGH translocations (fusion/break-apart probes) and 20% for del(17p), gain(1q), and del(1p).

We considered the baseline serum creatinine as the lowest value of serum creatinine available in the three months prior to diagnosis. The estimated glomerular filtration rate (eGFR) was calculated using the CKD-EPI equation [40]. CKD was defined according to the KDIGO guidelines as an estimated glomerular filtration rate below 60 mL/min/1.73 m^2^ for more than three months [41]. AKI was defined using the KDIGO creatinine criteria, which include a rise in serum creatinine of at least 0.3 mg/dL (26.5 μmol/L) within 48 h or an increase in serum creatinine to at least 1.5 times the baseline level, occurring or suspected to have occurred within the past seven days. Severity stages for AKI were also classified according to creatinine criteria: a rise of 1.5 to 2 times the baseline value was considered stage 1; a rise of 2 to 3 times the baseline value was considered stage 2; and a rise above 4 times the baseline value or the need for renal replacement therapy was considered stage 3 [24]. Moderate-to-severe AKI was considered when stage 2 or 3 was reached.

Heart disease was considered present when at least one of the following conditions was identified: coronary artery disease, congestive heart failure, myocardial infarction, left ventricular ejection fraction ≤ 50%, or valvular disease (excluding mitral prolapse).

Relapse-free survival was calculated in days until disease relapse for all patients. Overall survival was calculated in days until death from any cause for all patients. Progression to CKD was considered when an eGFR below 60 mL/min/1.73 m^2^ was recorded after two years from the diagnosis of multiple myeloma in survivors who did not present with CKD at the start of the study.

### 4.3. Statistical Methods

Categorical variables were described as frequencies (percentages) and quantitative data as medians (P25 = 25th percentile; P75 = 75th percentile). To evaluate the impact of high-risk cytogenetics on AKI, we first performed a univariable analysis to identify potential statistically significant factors using the Student’s *t*-test for continuous variables and the Chi-square test for categorical variables. Covariates selected for this analysis were based on clinical relevance and prior evidence suggesting their association with AKI.

Subsequently, we employed multivariable logistic regression to identify independent associations, adjusting for confounders that were either significant in the univariable analysis or considered clinically important. Variables that reached a significance threshold of *p* < 0.200 in the univariable analysis were retained for further modeling to account for potential confounding effects. To mitigate overfitting, we used backward stepwise regression to refine the final multivariable model, systematically removing non-significant covariates based on a *p*-value threshold of 0.05.

The impact of AKI on overall survival was evaluated using survival analysis for time until death from any cause with Cox proportional hazards regression models for both univariable and multivariable analyses. We checked the proportional hazards assumption by examining scaled Schoenfeld residuals.

To study the impact of AKI on disease-free survival, we applied survival analysis methods accounting for competing risks, specifically the Fine and Gray method [42] (which treats death as a competing risk), for both univariable and multivariable analyses. This approach is recommended by the European Group for Blood and Marrow Transplantation to appropriately model competing risks in survival data [43].

For assessing the progression to chronic kidney disease (CKD) after 2 years of diagnosis, we considered only patients who were free of CKD at baseline and remained alive at the 2-year follow-up. A two-proportion Z-test was used to assess differences in the proportion of patients developing CKD between those who experienced AKI versus those who did not.

All final multivariable models were selected using backward stepwise regression, starting with variables that achieved a significance level of *p* < 0.200 in the univariable analysis. The assumption of proportional hazards for the Cox model was verified using formal tests and graphical assessments of scaled Schoenfeld residuals. Hazard ratios, both crude and adjusted, were calculated along with their 95% confidence intervals (CIs), and a significance level of α = 0.05 was applied.

Data analysis was conducted using STATA for Windows (StataCorp, 2019. Stata Statistical Software: Release 16, College Station, TX, USA: StataCorp LLC.) and R software version 2017 (R Core Team, 2017). 

## Figures and Tables

**Table 1:** Patients’ baseline characteristics and multiple myeloma-related variables.

Patients’ Demographic Characteristics	Category	*n* (%) or P50 (P25–P75)
Age (years)		70.1 (60.8–78.7)
Gender	Male	64 (52.5)
Race	Caucasian	111 (90.9)
Chronic Kidney Disease		39 (32.0)
Hypertension		79 (64.8)
Diabetes Mellitus		28 (23.0)
Heart Disease		34 (27.9)
MM characteristics at presentation		
R-ISS Staging	I	31 (25.4)
	II	70 (57.4)
	III	21 (17.2)
Secondary Amyloidosis		4 (3.3)
Extramedullary Disease		26 (21.3)
Light Chain	L	55 (45.1)
	K	67 (54.9)
Isotype of Myeloma	Light chains	17 (13.9)
	IgA	29 (23.8)
	IgD	1 (0.8)
	IgG	74 (60.7)
	IgM	1 (0.8)
High Risk Cytogenetics		57 (46.7)
Hypercalcemia		18 (14.8)
AKI		45 (36.9)
FLC Ratio		1.8 (0.05–23.1)
dFLC (mg/L)		247.8 (48.9–1152.5)
M component (g/dL)		2.7 (1.1–4.3)
Albumin (g/dL)		3.7 (3.1–4.1)
β2 Microglobulin (mg/L)		4.76 (3.0–9.4)
LDH (U/L)		168 (138–211)
Plasmocytes in Bone Marrow (%)		39 (16–67)
Undergone treatments		
Proteasome Inhibitors		100 (81.9)
Autologous HSCT		38 (31.2)
Total		122 (100.0)

P50—median; P25—25th percentile; P75—75th percentile; R-ISS—International Staging System; MM—multiple myeloma; L—lambda; K—kappa; Ig—Immunoglobulin; AKI—acute kidney injury; FLC –free light chains; dFLC—difference in free light chains; LDH—lactate dehydrogenase.

**Table 2:** Univariable analysis for AKI in newly diagnosed multiple myeloma patients.

Variables	AKI n = 45	Non AKI n = 77	*p*-Value
Age	69.9 (63.2–76.6)	71.8 (60.3–79.4)	0.524
Gender (Male)—*n* (%)	27 (60.0)	37 (48.1)	0.202
Race (Caucasian)—*n* (%)	42 (93.3)	69 (89.6)	0.488
Chronic Kidney Disease—*n* (%)	25 (55.6)	14 (18.2)	<0.001
Hypertension—*n* (%)	33 (73.3)	46 (59.7)	0.129
Diabetes Mellitus—*n* (%)	13 (28.9)	15 (19.5)	0.233
Heart Disease—*n* (%)	23 (51.1)	11 (14.3)	0.519
Light Chain (Reference L)*—n* (%)	26 (57.8)	29 (37.7)	0.031
Hypercalcemia—*n* (%)	13 (28.9)	5 (6.5)	0.001
Secondary Amyloidosis—*n* (%)	2 (4.4)	2 (2.6)	0.580
Extramedullary Disease—*n* (%)	6 (13.3)	20 (26.0)	0.100
High-Risk Cytogenetics—*n* (%)	28 (62.2)	29 (37.7)	0.009
FLC Ratio	0.2 (0.01–36)	2.12 (0.1–20.9)	0.398
dFLC—(mg/L)	837.8 (284.6–3501)	127.6 (22.3–491.8)	<0.001
M component (g/dL)	2.6 (0.4–4.9)	2.6 (1.5–3.75)	0.294
Albumin (g/dL)	3.4 (3.0–4.1)	3.8 (3.2–4.1)	0.433
β2 Microglobulin (mg/L)	11.3 (6.35–18)	3.3 (2.6–4.6)	<0.001
LDH (U/L)	185 (140–223)	159.5 (136–193)	0.063
Plasmocytes in Bone Marrow (%)	51 (35–77)	30 (12–62)	0.001

L—lambda; K—kappa; Ig—immunoglobulin; FLC—free light chains; LDH—lactate dehydrogenase.

**Table 3:** Multivariable model for AKI in newly diagnosed multiple myeloma patients.

Variables	Odds Ratio Estimate	95% Confidence Interval		*p*-Value
		Lower Limit	Upper Limit	
Chronic Kidney Disease	9.14	2.92	18.65	<0.001
Light Chain (Reference L)	3.70	1.31	6.45	0.013
Hypercalcemia	3.17	1.50	5.36	0.001
High-risk Cytogenetics	3.32	1.17	6.40	0.024
dFLC	1.01	1.00	1.02	0.015
Bone Marrow Plasmocyte Percentage	1.02	1.01	1.04	0.010

L—lambda; FLC—free light chains.

**Table 4:** Cox proportional hazards model regression. Univariable analysis for mortality.

Variables	Hazards Ratio Estimate	95% Confidence Interval		*p*-Value
		Lower Limit	Upper Limit	
Age	1.05	1.01	1.09	0.011
Gender (Reference: Male)	0.78	0.38	1.57	0.484
Race (Reference: Caucasian)	1.19	0.36	3.90	0.778
Chronic Kidney Disease	2.59	1.28	5.23	0.008
Hypertension	0.83	0.40	1.71	0.613
Diabetes Mellitus	2.06	0.99	4.30	0.055
Heart Disease	2.60	1.29	5.28	0.008
Hypercalcemia	1.42	0.58	3.45	0.444
Secondary Amyloidosis	3.32	1.50	6.00	0.049
Extramedullary Disease	1.15	0.49	2.67	0.748
Light Chain (Reference L)	0.90	0.44	1.84	0.777
dFLC (mg/L)	1.01	1.00	1.01	0.028
M Component (mg/dL)	0.92	0.77	1.11	0.384
Albumin (g/dL)	0.96	0.57	1.59	0.865
β2 Microglobulin (mg/L)	1.06	1.02	1.10	0.003
LDH (U/L)	1.00	1.00	1.001	0.220
Plasmocytes in Bone Marrow (%)	1.01	1.00	1.02	0.202
High-risk Cytogenetics	4.21	1.61	9.82	0.003
AKI	2.16	1.06	4.38	0.033
Proteasome inhibitors	0.35	0.16	0.75	0.007
Autologous HSCT	0.47	0.22	1.02	0.057
Relapse	1.65	0.63	4.29	0.308

FLC—free light chains; LDH—lactate dehydrogenase; AKI—acute kidney injury; HSCT—Hematopoietic Stem Cell Transplant.

**Table 5:** Cox proportional hazards model regression. Multivariable analysis for mortality.

Variables	Hazard Ratio Estimate	95% Confidence Interval		*p*-Value
		Lower Limit	Upper Limit	
Diabetes Mellitus	1.99	1.59	4.44	0.026
Heart Disease	3.70	1.75	7.83	0.001
Autologous HSCT	0.15	0.05	0.51	0.002
High-Risk Cytogenetics	3.33	1.13	9.76	0.029
AKI	2.71	1.18	6.23	0.019

HSCT—Hematopoietic Stem Cell Transplant; AKI—acute kidney injury.

## Data Availability

The data underlying this article will be shared on reasonable request to the corresponding author.

## Supplementary Materials

The following supporting information can be downloaded at: https://www.mdpi.com/article/10.3390/ijms26136108/s1.
