# Supplementary material for: The Role of High-Risk Cytogenetics in Acute Kidney Injury of Newly Diagnosed Multiple Myeloma: A Cohort Study
Source: Int J Mol Sci. 2025 Jun 25;26(13):6108. doi: 10.3390/ijms26136108 (PMC12249657; doi:10.3390/ijms26136108)
Supplement: Supplementary file 1 [file ijms-26-06108-s001.zip › ijms-3660535-supplementary.pdf]

Supplementary Materials: STROBE (Strengthening The Reporting of OBservational Studies in Epidemiology) Checklist

Section and Item	Item No.	Recommendation	Reported on Page No.
**Title and Abstract**	1	(*a*) Indicate the study's design with a commonly used term in the title or the abstract	Page 1, see title
		(*b*) Provide in the abstract an informative and balanced summary of what was done and what was found	Page 1, see abstract
**Introduction**			
Background/Rationale	2	Explain the scientific background and rationale for the investigation being reported	Pages 1-2, Paragraph 1-4: provides background. Paragraph 4: provides rationale
Objectives	3	State specific objectives, including any prespecified hypotheses	Page 2, Paragraph 5
**Methods**			
Study Design	4	Present key elements of study design early in the paper	Page 9, Section 4.1, Paragraph 1
Setting	5	Describe the setting, locations, and relevant dates, including periods of recruitment, exposure, follow-up, and data collection	Page 9, Section 4.1, Paragraph 1-4
Participants	6	(*a*) *Cohort study*—Give the eligibility criteria, and the sources and methods of selection of participants. Describe methods of follow-up	Page 9, Section 4.1, Paragraph 1-2
		(*b*) *Cohort study*—For matched studies, give matching criteria and number of exposed and unexposed	not applicable
Variables	7	Clearly define all outcomes, exposures, predictors, potential confounders, and effect modifiers. Give diagnostic criteria, if applicable	Page 9-10, Section 4.2 Paragraph 1-4

Section and Item	Item No.	Recommendation	Reported on Page No.
Data Sources/ Measurement	8*	For each variable of interest, give sources of data and details of methods of assessment (measurement). Describe comparability of assessment methods if there is more than one group	Page 9, Section 4.1, Paragraph 3<br>Pages 9-10, Section 4.2, Paragraph 1-4
Bias	9	Describe any efforts to address potential sources of bias	Page 10, Section 4.3, Paragraphs 2-6
Study Size	10	Explain how the study size was arrived at	Present in the Results Section: Page 2 and in Figure 1.
Quantitative Variables	11	Explain how quantitative variables were handled in the analyses. If applicable, describe which groupings were chosen and why	Page 10, Section 4.3, Paragraph 1
Statistical Methods	12	(*a*) Describe all statistical methods, including those used to control for confounding	Pages 10-11, Section 4.3, Paragraph 1-6
		(*b*) Describe any methods used to examine subgroups and interactions	Page 10, Section 4.3 Paragraph 2 and 4
		(*c*) Explain how missing data were addressed	Page 9, Section 4.1, Paragraph 3
		(*d*) *Cohort study*—If applicable, explain how loss to follow-up was addressed	Page 9, Section 4.1, Paragraph 4
		(*e*) Describe any sensitivity analyses	Page 10, Section 4.3, Paragraphs 2-6
**Results**			
Participants	13*	(a) Report numbers of individuals at each stage of study—eg numbers potentially eligible, examined for eligibility, confirmed eligible, included in the study, completing follow-up, and analysed	Page 2, Results section, Paragraph 1
		(b) Give reasons for non-participation at each stage	Page 2, Results section, Paragraph 1
		(c) Consider use of a flow diagram	Page 3, Figure 1.
Descriptive Data	14*	(a) Give characteristics of study participants (eg demographic, clinical, social) and information on exposures and potential confounders	Page 3, Table 1.
		(b) Indicate number of participants with missing data for each variable of interest	See Materials and Methods, Section 4.1; Page 9, Paragraph 3
		(c) Cohort study—Summarise follow-up time (eg, average and total amount)	See Materials and Methods, Section 4.1;

			Page 9, Paragraph 4
Outcome Data	15*	Cohort study—Report numbers of outcome events or summary measures over time	Pages 4-6, Section “AKI at diagnosis” - Paragraph 1 Section “The impact of AKI in overall survival”- Paragraph 1 Section “The impact of AKI on relapse free survival” Paragraph 1 Section “The impact of AKI in the progression to CKD”

Section and Item	Item No.	Recommendation	Reported on Page No.
Main Results	16	(*a*) Give unadjusted estimates and, if applicable, confounder-adjusted estimates and their precision (eg, 95% confidence interval). Make clear which confounders were adjusted for and why they were included	Pages 4-6, Table 2-5
		(*b*) Report category boundaries when continuous variables were categorized	Page 3, Table 1; Page 4, Table 3, Page 5, Table 4 Page 6, Table 5
		(*c*) If relevant, consider translating estimates of relative risk into absolute risk for a meaningful time period	—
Other Analyses	17	Report other analyses done—eg analyses of subgroups and interactions, and sensitivity analyses	Page 10, Section 4.3, Paragraphs 2-5
**Discussion**			
Key Results	18	Summarise key results with reference to study objectives	Page 7, Paragraph 2-3; 5; Page 8, Paragraph 2 and 4
Limitations	19	Discuss limitations of the study, taking into account sources of potential bias or imprecision. Discuss both direction and magnitude of any potential bias	Pages 8, Paragraph 6
Interpretation	20	Give a cautious overall interpretation of results considering objectives, limitations, multiplicity of analyses, results from similar studies, and other relevant evidence	Page 7, Paragraphs 3 and 6; Page 8, Paragraph 2
Generalisability	21	Discuss the generalisability (external validity) of the study results	Page 8, Paragraph 6
**Other Information**			
Funding	22	Give the source of funding and the role of the funders for the present study and, if applicable, for the original study on which the present article is based	Page 11, Section “Funding”
